# Antibiotic exposure and indication-specific corticosteroid use differentially modulate outcomes of immune checkpoint inhibitor therapy in hepatobiliary malignancies

**DOI:** 10.3389/fimmu.2026.1873839

**Published:** 2026-06-29

**Authors:** Jiabao Yang, Wenjuan Wang, Min Jiao, Zhekang Zhang, Shuwei Lei, Ning Lan, Min Chen, Zirui Wang, Beina Hui, Tasneem Ibrahim Dawalbait Bakhat, Juan Ren

**Affiliations:** 1 Department of Radiotherapy, The First Affiliated Hospital of Xi’an Jiaotong University, Xi’an, China; 2 Department of Medical Oncology, The First Affiliated Hospital of Xi’an Jiaotong University, Xi’an, China; 3 Department of Medicine, Xi′an Jiaotong University, Xi′an, China

**Keywords:** antibiotics, corticosteroids, hepatobiliary malignancies, immune checkpoint inhibitors, microbiome, survival analysis, tumor microenvironment

## Abstract

**Background:**

Concomitant medications may influence the tumor–immune microenvironment and potentially affect the efficacy of immune checkpoint inhibitors (ICIs), yet their impact in hepatobiliary malignancies remains poorly defined. We evaluated the associations of antibiotic (ATB) exposure and indication-specific corticosteroid (CS) use with clinical outcomes in hepatocellular carcinoma (HCC) and cholangiocarcinoma (CCA).

**Methods:**

In this retrospective cohort of 759 ICI-treated patients (511 HCC, 248 CCA), patients were stratified into four groups: no exposure (None, n=251), ATB only (n=135), CS only (n=183), and combined exposure (Both, n=190). ATB exposure was defined as systemic use within ±30 days of ICI initiation, and CS exposure as ≥10 mg prednisone-equivalent daily for ≥3 consecutive days. Inverse probability of treatment weighting (IPTW) and multivariable Cox models were used to adjust for confounding. Additional baseline-only sensitivity analyses restricted exposure to the 30 days before ICI initiation to reduce potential time-related bias.

**Results:**

After IPTW adjustment, baseline covariates were well balanced across exposure groups. Combined ATB and CS exposure was associated with significantly worse overall survival (OS) (adjusted HR 3.09, 95% CI 2.31–4.13; p<0.001) and progression-free survival (PFS) compared with no exposure. Objective response rates were comparable across groups (p=0.20), suggesting a greater association with impaired response durability rather than initial tumor shrinkage. Among CS-treated patients, corticosteroid use for immune-related adverse event (irAE) management was associated with improved OS compared with non-irAE indications (p<0.01). Landmark and baseline-only sensitivity analyses demonstrated generally consistent findings.

**Conclusions:**

Antibiotic exposure and corticosteroid use for non-irAE indications were associated with inferior survival outcomes in ICI-treated hepatobiliary malignancies. These findings suggest that concomitant medication exposure may influence the durability of immunotherapy responses in real-world clinical settings. In contrast, corticosteroid use for irAEs was not associated with compromised outcomes, supporting the importance of context-specific corticosteroid administration during immunotherapy. Clinically, these findings highlight the importance of judicious antibiotic use and careful consideration of corticosteroid indications during ICI treatment.

## Introduction

Immune checkpoint inhibitors (ICIs) targeting the programmed cell death-1 (PD-1)/PD-L1 axis have transformed the treatment of advanced hepatobiliary malignancies, including hepatocellular carcinoma (HCC) and cholangiocarcinoma (CCA) (1–3). Landmark trials, such as IMbrave150, CheckMate 040, and HIMALAYA for HCC, and TOPAZ-1 for CCA, have established ICI-based combinations as first-line standard of care (4–7). Despite these advances, a substantial proportion of patients fail to achieve durable responses, highlighting the need to identify clinical factors that influence immune checkpoint inhibitor efficacy in real-world hepatobiliary cancer populations (8, 9).

In clinical practice, patients with hepatobiliary malignancies frequently receive concomitant medications, most notably broad-spectrum antibiotics (ATBs) and corticosteroids (CSs). ATBs are often prescribed for biliary tract infections or spontaneous bacterial peritonitis, whereas CSs are used both to manage immune-related adverse events (irAEs) and to palliate cancer-related symptoms such as pain or peritumoral edema. Evidence from other solid tumors suggests that ATB exposure may impair ICI efficacy by altering the gut-liver axis and modulating systemic immune responses (10, 11). Likewise, the immunosuppressive properties of CSs raise concerns that they may counteract the T-cell activation required for effective immunotherapy (12).

The impact of these medications in hepatobiliary cancers remains unclear. Previous studies have been limited by small sample sizes, heterogeneous populations, and, importantly, a lack of distinction between steroid indications (13–15). Baseline steroid use for symptom control is generally linked to poor prognosis, but whether steroids administered for irAE management exert similar detrimental effects is uncertain. Moreover, the potential synergistic impact of combined ATB and CS exposure—a common clinical scenario—has not been thoroughly examined in large hepatobiliary cohorts.

Retrospective analyses in this field are often confounded by immortal time and selection biases, as patients with more aggressive disease are more likely to receive supportive medications (16). To provide robust evidence, it is essential to employ statistical approaches such as inverse probability of treatment weighting (IPTW) to balance baseline characteristics and isolate the independent effects of these drugs.

In this study, we evaluated the impact of ATB and indication-specific CS use on clinical outcomes in a large cohort of 759 patients with advanced hepatobiliary malignancies. Using IPTW-adjusted models and landmark analyses, we aimed to clarify both the independent and synergistic roles of these concomitant medications, and to determine whether the negative impact of CS is dependent on the clinical indication for its use.

## Materials and methods

### Study design and population

This retrospective cohort study included patients with hepatocellular carcinoma (HCC) and cholangiocarcinoma (CCA) treated with immune checkpoint inhibitors between January 1, 2020 and January 31, 2025 at the First Affiliated Hospital of Xi’an Jiaotong University. We initially screened 4894 patients with histologically or cytologically confirmed hepatobiliary malignancies who received ICIs during this period.

Patients were eligible for inclusion if they met all of the following criteria: (I) were ≥18 years old; (II) had advanced, unresectable hepatocellular carcinoma (HCC) or cholangiocarcinoma (CCA); (III) received at least two cycles of ICI-based systemic therapy; and (IV) had at least one baseline and one follow-up radiological assessment (CT or MRI) for efficacy evaluation.

**Figure 1:**
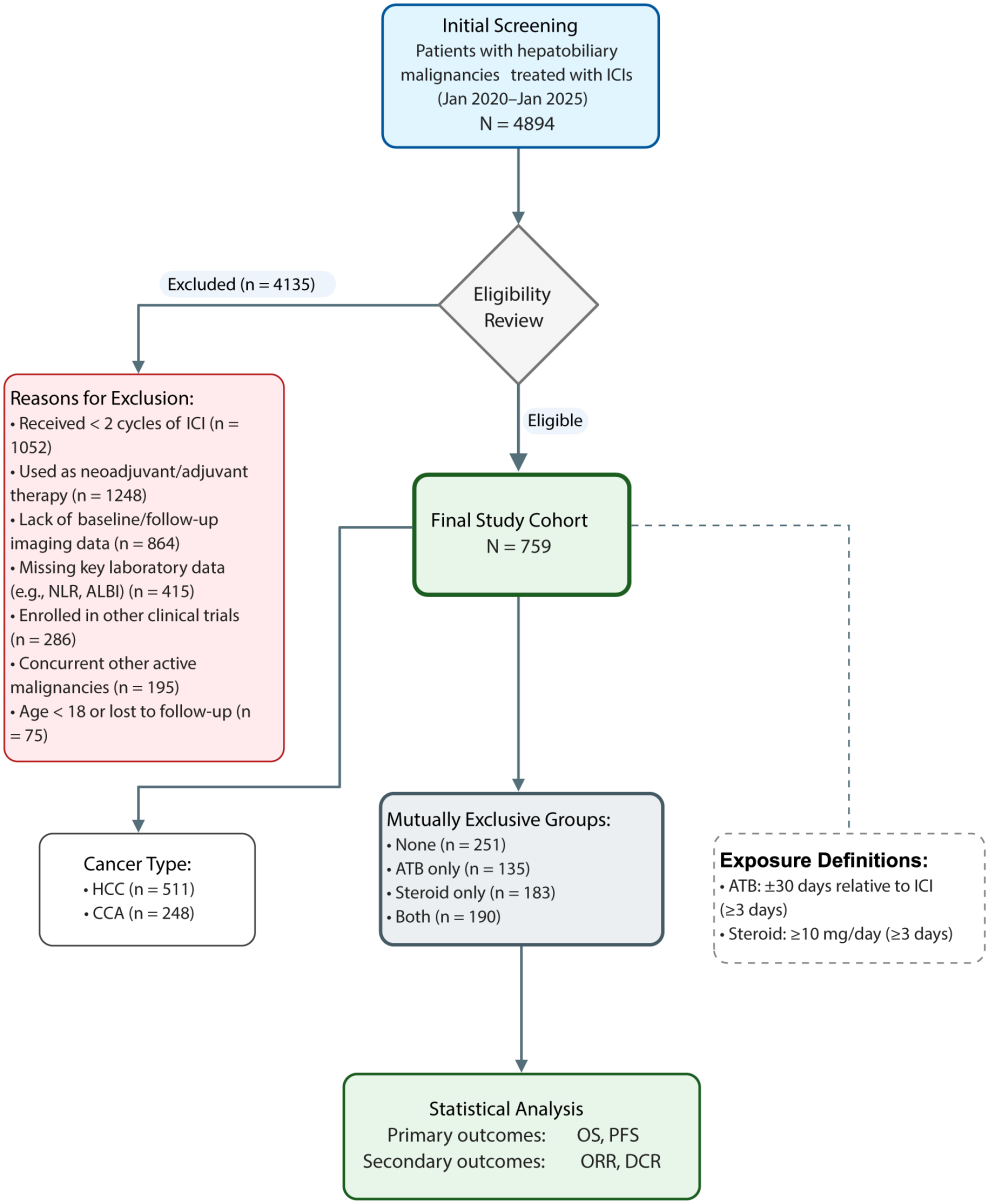
Flowchart of the study population. A total of 4894 patients with hepatobiliary malignancies treated with immune checkpoint inhibitors (ICIs) were screened, of whom 759 were included in the final analysis. Patients were categorized into four groups according to exposure to antibiotics (ATB) and corticosteroids. ALBI, albumin–bilirubin; ATB, antibiotics; CCA, cholangiocarcinoma; DCR, disease control rate; HCC, hepatocellular carcinoma; ICI, immune checkpoint inhibitor; NLR, neutrophil-to-lymphocyte ratio; ORR, objective response rate; OS, overall survival; PFS, progression-free survival.

We excluded 4135 patients according to the following criteria: (I) ICI used in neoadjuvant or adjuvant settings (n=1248); (II) received <2 cycles of ICI (*n* = 1052); (III) lack of baseline or follow-up imaging data (*n* = 864); (IV) missing key laboratory or clinical data (*n* = 415); (V) enrollment in interventional clinical trials (*n* = 286); (VI) concurrent other active malignancies (*n* = 195); or (VII) lost to follow-up (*n* = 75). Ultimately, 759 patients (511 HCC and 248 CCA) were included in the final analysis (**Figure 1**).

The study was approved by the Institutional Review Board of the First Affiliated Hospital of Xi’an Jiaotong University and conducted in accordance with the Declaration of Helsinki (17). Given its retrospective design, the requirement for informed consent was waived.

### Definitions of medication exposure

Medication exposure was assessed within a 60-day window (-30 to +30 days relative to the first dose of ICI). This timeframe was selected based on previous studies suggesting that the gut microbiome and host immune environment may be particularly susceptible to pharmacological perturbation during the initiation phase of immunotherapy (11, 18). A sensitivity analysis using a baseline-only exposure window (−30 to 0 days before ICI initiation) was additionally performed to reduce potential time-related bias.

Antibiotic (ATB) exposure was defined as the systemic administration (oral or intravenous) of broad-spectrum antibiotics for ≥3 consecutive days within the defined window. Corticosteroid (CS) exposure was defined as a cumulative prednisone-equivalent dose ≥10 mg/day for ≥3 consecutive days (12). Based on exposure status, patients were categorized into four mutually exclusive groups: None (no exposure, n=251), ATB only (n=135), CS only (n=183), and Both (combined exposure, n=190). Because of the retrospective nature of the study, detailed time-varying modeling of medication sequence, cumulative exposure intensity, and dynamic overlap between antibiotics and corticosteroids was not feasible.

### Indication for corticosteroid use

All electronic medical records were reviewed to classify the primary indication for CS initiation into two categories:

1. irAE-related: CS administered to manage immune-related adverse events (e.g., immune-mediated hepatitis, pneumonitis, colitis) (19, 20).
2. Symptomatic management (Non-irAE): CS administered to alleviate cancer-related symptoms(e.g., pain, peritumoral edema, cancer-related cachexia).

Patients receiving low-dose or single-dose CS for chemotherapy premedication who did not meet the exposure threshold (prednisone-equivalent ≥10 mg/day for ≥3 days) were classified as non-exposed.

### Outcome measures

The primary endpoints were overall survival (OS) and progression−free survival (PFS). OS was defined as the time from ICI initiation to death from any cause. PFS was defined as the time from ICI initiation to first documented disease progression or death. Tumor response was evaluated according to RECIST v1.1, which remains the most commonly used response assessment criterion in retrospective real-world studies of hepatobiliary malignancies receiving immunotherapy (21). Secondary endpoints included objective response rate (ORR) and disease control rate (DCR), both assessed according to RECIST v1.1 criteria. Although immune-specific criteria such as iRECIST or modified RECIST (mRECIST) may better capture atypical response patterns during immunotherapy, standardized confirmatory imaging assessments required for these criteria were not consistently available in this retrospective cohort.

### Statistical analysis

Baseline characteristics were compared using the chi-square or Fisher’s exact test for categorical variables and the Kruskal–Wallis test for continuous variables.

To control for confounding and baseline imbalances, inverse probability of treatment weighting (IPTW) was applied using propensity scores estimated from a multinomial logistic regression model (22, 23). Covariates included age, cancer type (HCC vs. CCA), TNM stage, ECOG performance status, ALBI grade, LIPI score, baseline neutrophil-to-lymphocyte ratio (NLR), and treatment regimen (24, 25). Stabilized weights were applied to reduce the influence of extreme observations and improve model stability. Covariate balance before and after weighting was assessed using standardized mean differences (SMDs), with values <0.1 indicating adequate balance. Weight distributions and propensity score overlap were visually inspected to evaluate common support, positivity assumptions, and weighting stability (**Supplementary Figure 1, S2, and S3**; **Supplementary Methods 1**).

Survival outcomes were analyzed using the Kaplan–Meier method and compared with the log-rank test. Multivariable Cox proportional hazards models were used to estimate hazard ratios (HRs) and 95% confidence intervals (CIs).

To reduce immortal time bias, a landmark analysis was performed at 6 months after ICI initiation. Only patients who were alive and event-free at the landmark time point were included in the landmark cohort (**Supplementary Methods 2**) (26). In addition, a sensitivity analysis was performed using a baseline-only exposure definition restricted to the 30 days before immune checkpoint inhibitor initiation (−30 to 0 days) to further minimize potential immortal time bias and reverse causality related to post-treatment medication exposure.

All statistical analyses were conducted using R software (version 4.3.2), and a two-sided p < 0.05 was considered statistically significant. Additional methodological details are provided in the **Supplementary Methods 3**.

## Results

### Patient characteristics and covariate balance

Between January 2020 and January 2025, 759 eligible patients with advanced hepatobiliary malignancies (511 HCC and 248 CCA) were included in the analysis (**Figure 1**). Baseline characteristics are summarized in **Table 1**. Significant differences were observed across the four exposure groups for cancer type (p<0.001), liver function (ALBI grade, p=0.002), systemic inflammation (LIPI score and baseline NLR, both p<0.001), and treatment regimen (p<0.001). Patients in the ATB only and Both groups were more likely to receive ICI combined with chemotherapy (43.0% and 26.3%, respectively) compared with the None group (21.9%). Overall, the Both group exhibited the least favorable clinical profile, with a higher median baseline NLR (7.30 [IQR 5.30–10.30]).

**Table 1:** Baseline clinical and demographic characteristics of the study population.

Characteristics	level	Overall	None	ATB only	Steroid only	Both	P
**n**		759	251	135	183	190	
**Age (median [IQR])**		61.00[51.00, 71.00]	62.00[51.00, 71.00]	61.00[51.50, 68.00]	63.00[55.00, 71.00]	59.00[49.00, 69.00]	0.286
**Sex (%)**	Female	347 (45.7)	114 (45.4)	57 (42.2)	89 (48.6)	87 (45.8)	0.729
	Male	412 (54.3)	137 (54.6)	78 (57.8)	94 (51.4)	103 (54.2)	
**Cancer type (%)**	HCC	511 (67.3)	182 (72.5)	59 (43.7)	148 (80.9)	122 (64.2)	<0.001
	CCA	248 (32.7)	69 (27.5)	76 (56.3)	35 (19.1)	68 (35.8)	
**Stage merged (%)**	Stage III	348 (45.8)	119 (47.4)	60 (44.4)	85 (46.4)	84 (44.2)	0.900
	Stage IV	411 (54.2)	132 (52.6)	75 (55.6)	98 (53.6)	106 (55.8)	
**Extrahepatic Metastasis (%)**	No	348 (45.8)	119 (47.4)	60 (44.4)	85 (46.4)	84 (44.2)	0.900
	Yes	411 (54.2)	132 (52.6)	75 (55.6)	98 (53.6)	106 (55.8)	
**ECOG PS (%)**	0-1	185 (24.4)	68 (27.1)	23 (17.0)	51 (27.9)	43 (22.6)	0.091
	≥2	574 (75.6)	183 (72.9)	112 (83.0)	132 (72.1)	147 (77.4)	
**Child-Pugh score (%)**	A	284 (37.4)	92 (36.7)	56 (41.5)	66 (36.1)	70 (36.8)	0.757
	B	475 (62.6)	159 (63.3)	79 (58.5)	117 (63.9)	120 (63.2)	
**ALBI Score (%)**	Grade 1	282 (37.2)	120 (47.8)	48 (35.6)	55 (30.1)	59 (31.1)	0.002
	Grade 2	311 (41.0)	82 (32.7)	55 (40.7)	89 (48.6)	85 (44.7)	
	Grade 3	166 (21.9)	49 (19.5)	32 (23.7)	39 (21.3)	46 (24.2)	
**LIPI Score (%)**	Good	254 (33.5)	110 (43.8)	33 (24.4)	59 (32.2)	52 (27.4)	<0.001
	Intermediate	289 (38.1)	97 (38.6)	50 (37.0)	78 (42.6)	64 (33.7)	
	Poor	216 (28.5)	44 (17.5)	52 (38.5)	46 (25.1)	74 (38.9)	
**Line of Therapy (%)**	1st line	357 (47.0)	133 (53.0)	61 (45.2)	85 (46.4)	78 (41.1)	0.089
	2nd+ line	402 (53.0)	118 (47.0)	74 (54.8)	98 (53.6)	112 (58.9)	
**Treatment regimen (%)**	PD-1 mono	107 (14.1)	39 (15.5)	17 (12.6)	26 (14.2)	25 (13.2)	<0.001
	PD-1+TKI	386 (50.9)	131 (52.2)	48 (35.6)	112 (61.2)	95 (50.0)	
	PD-1+Chemo	188 (24.8)	55 (21.9)	58 (43.0)	25 (13.7)	50 (26.3)	
	PD-1+Radio	78 (10.3)	26 (10.4)	12 (8.9)	20 (10.9)	20 (10.5)	
**Baseline NLR (median [IQR])**		5.20 [3.60, 6.70]	3.60 [2.70, 4.55]	5.30 [4.50, 6.65]	5.30 [3.60, 6.30]	7.30 [5.30, 10.30]	<0.001

Data are presented as n (%) or median [interquartile range].

Continuous variables were compared using the Kruskal–Wallis test.

Categorical variables were compared using the Chi-square or Fisher’s exact test.

ALBI, albumin-bilirubin; ATB, antibiotics; CCA, cholangiocarcinoma; CS, corticosteroids; ECOG PS, Eastern Cooperative Oncology Group performance status; HCC, hepatocellular carcinoma; LIPI, Lung Immune Prognostic Index; NLR, neutrophil-to-lymphocyte ratio.

The bold values indicate statistical significance (P < 0.05).

To mitigate potential confounding, IPTW weighting was applied. Post-weighting diagnostics demonstrated substantially improved covariate balance across exposure groups, with all standardized mean differences reduced to <0.1 (**Supplementary Figure 1**). In addition, stabilized weight distribution plots showed no evidence of marked instability, while propensity score overlap plots demonstrated acceptable common support across exposure groups (**Supplementary Figure 2, S3**).

### Impact of medication exposure on survival outcomes

**Figure 2:**
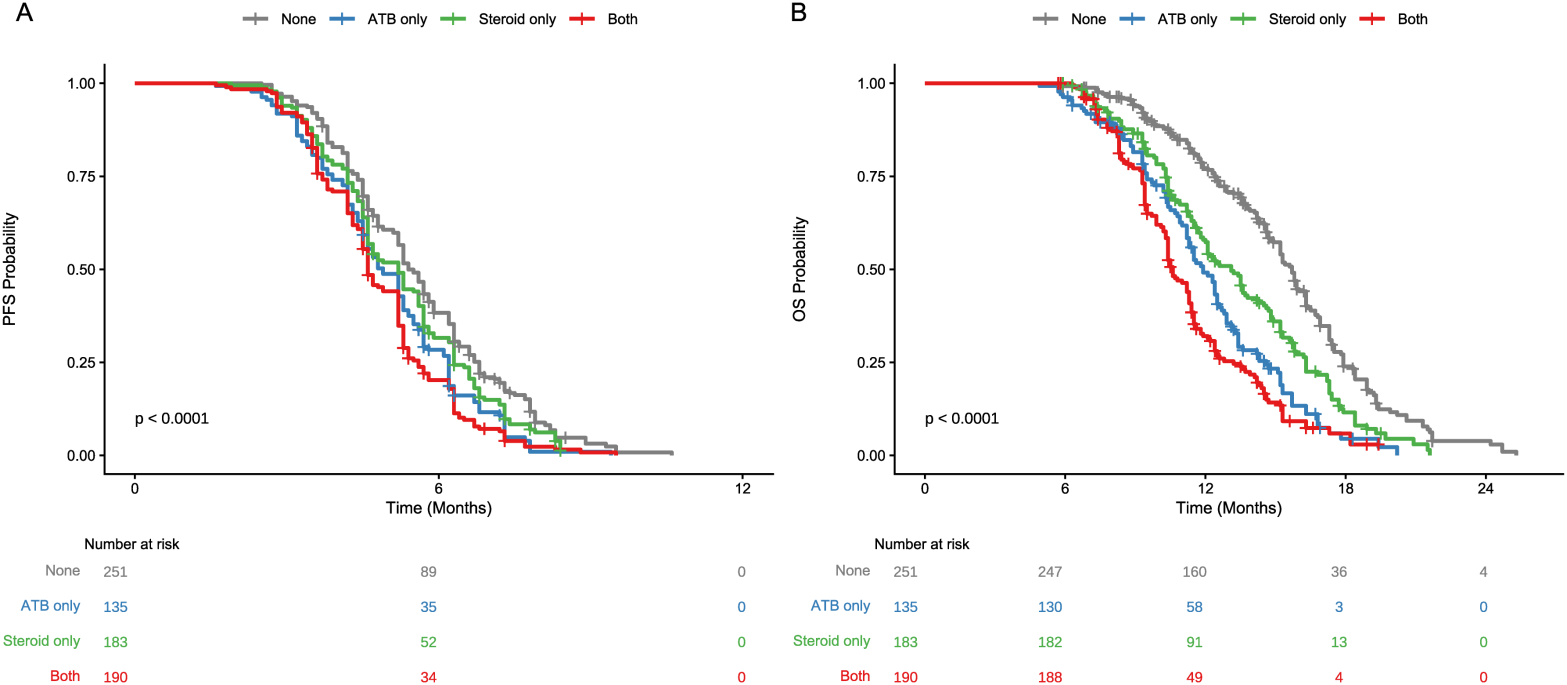
Kaplan–Meier curves for progression-free survival (PFS) and overall survival (OS) in the original cohort. **(A)** PFS and **(B)** OS stratified by the four medication exposure groups. Survival curves were compared using the log-rank test. ATB, antibiotics; OS, overall survival; PFS, progression-free survival.

In the unadjusted cohort, medication exposure was associated with significantly shorter survival (**Figure 2**). Median overall survival (OS) decreased stepwise from the None group to the Both group (p<0.0001; **Figure 2B**). Similar stepwise deteriorations were observed for PFS (p<0.0001; **Figure 2A**). After IPTW adjustment, the associations between medication exposure and both PFS and OS remained statistically significant (PFS: p<0.0001, **Supplementary Figure 4A**; OS: p<0.0001; **Supplementary Figure 4B**), suggesting that the observed survival differences were not fully explained by measured baseline clinical characteristics.

Sensitivity analyses using a baseline-only exposure definition (−30 to 0 days before immune checkpoint inhibitor initiation) demonstrated generally consistent associations between medication exposure and survival outcomes, although the effect sizes were attenuated compared with the primary analysis (**Supplementary Figure 5**; **Supplementary Table 1**).

### Tumor response and clinical efficacy

**Figure 3:**
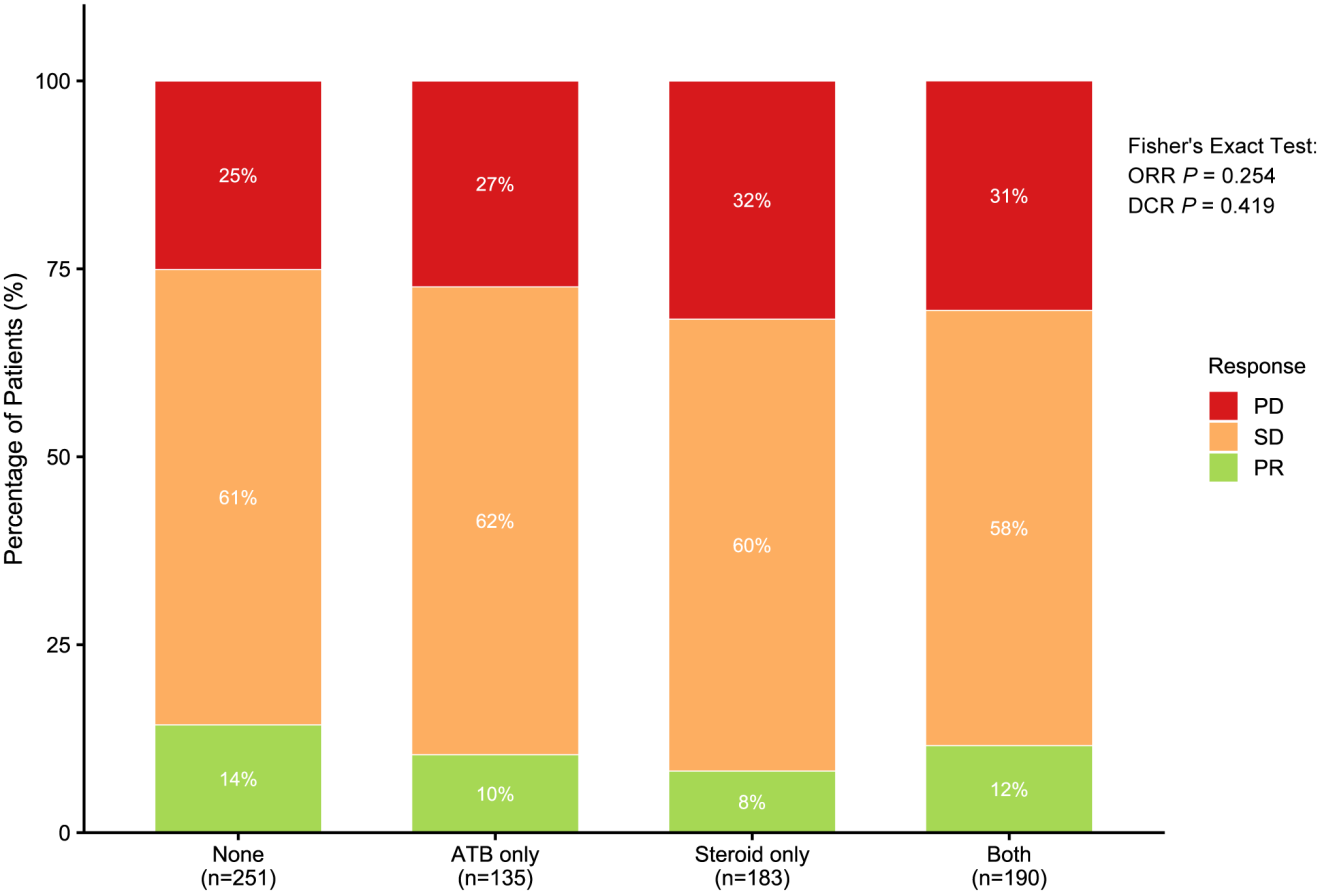
Best overall response according to medication exposure. Distribution of best overall response (PR, SD, and PD) across the four groups. *p*-values for objective response rate (ORR) and disease control rate (DCR) were calculated using Fisher’s exact test. PD, progressive disease; PR, partial response; SD, stable disease.

The objective response rate (ORR) was 14.3% in the None group, 10.4% in the ATB only group, 8.2% in the CS only group, and 11.6% in the Both group, with no statistically significant difference (p=0.254; **Figure 3**). Similarly, no significant between-group difference was observed in the disease control rate (DCR) (None: 74.9% vs. Both: 69.5%, p=0.419) (**Supplementary Table 2**). These findings suggest that concomitant medication exposure may have a greater association with long-term survival outcomes than with initial radiographic tumor response (10, 11).

### Independent prognostic factors

**Figure 4:**
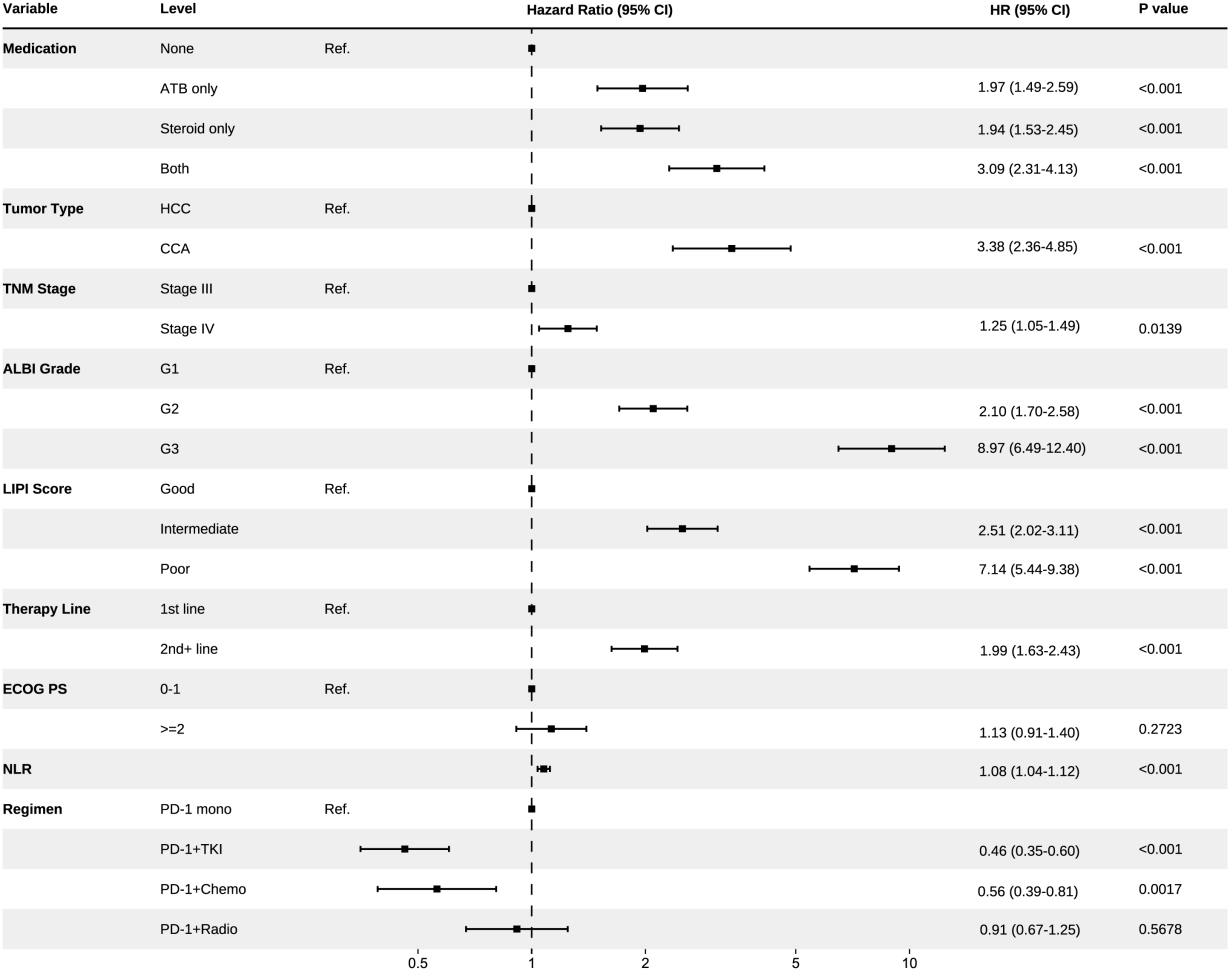
Multivariable Cox proportional hazards analysis for overall survival. Forest plot of hazard ratios (HRs) and 95% confidence intervals (CIs) for overall survival associated with medication exposure and clinical covariates, derived from multivariable Cox models. Detailed results for overall survival and progression-free survival are provided in **Supplementary Table 2**. CI, confidence interval; HR, hazard ratio; LIPI, Lung Immune Prognostic Index.

In multivariable Cox regression analysis, medication exposure was identified as an independent predictor of poorer OS (**Figure 4**; **Supplementary Table 3**). Compared with the None group, combined ATB and CS exposure (Both group) was associated with a more than threefold increase in mortality risk (Adjusted HR 3.09; 95% CI 2.31-4.13; *p* < 0.001). ATB only (Adjusted HR 1.97; 95% CI 1.49-2.59; *p* < 0.001) and CS only (Adjusted HR 1.94; 95% CI 1.53-2.45; *p* < 0.001) also remained significant risk factors. Other independent prognostic factors for OS included CCA subtype, ALBI Grade 3, and poor LIPI score (**Supplementary Table 3**). For PFS, the Both group also demonstrated a significantly higher risk of progression (HR 1.30; 95% CI 1.02–1.65; *p* = 0.037; **Supplementary Table 3**).

### Differential impact by steroid indication

**Figure 5:**
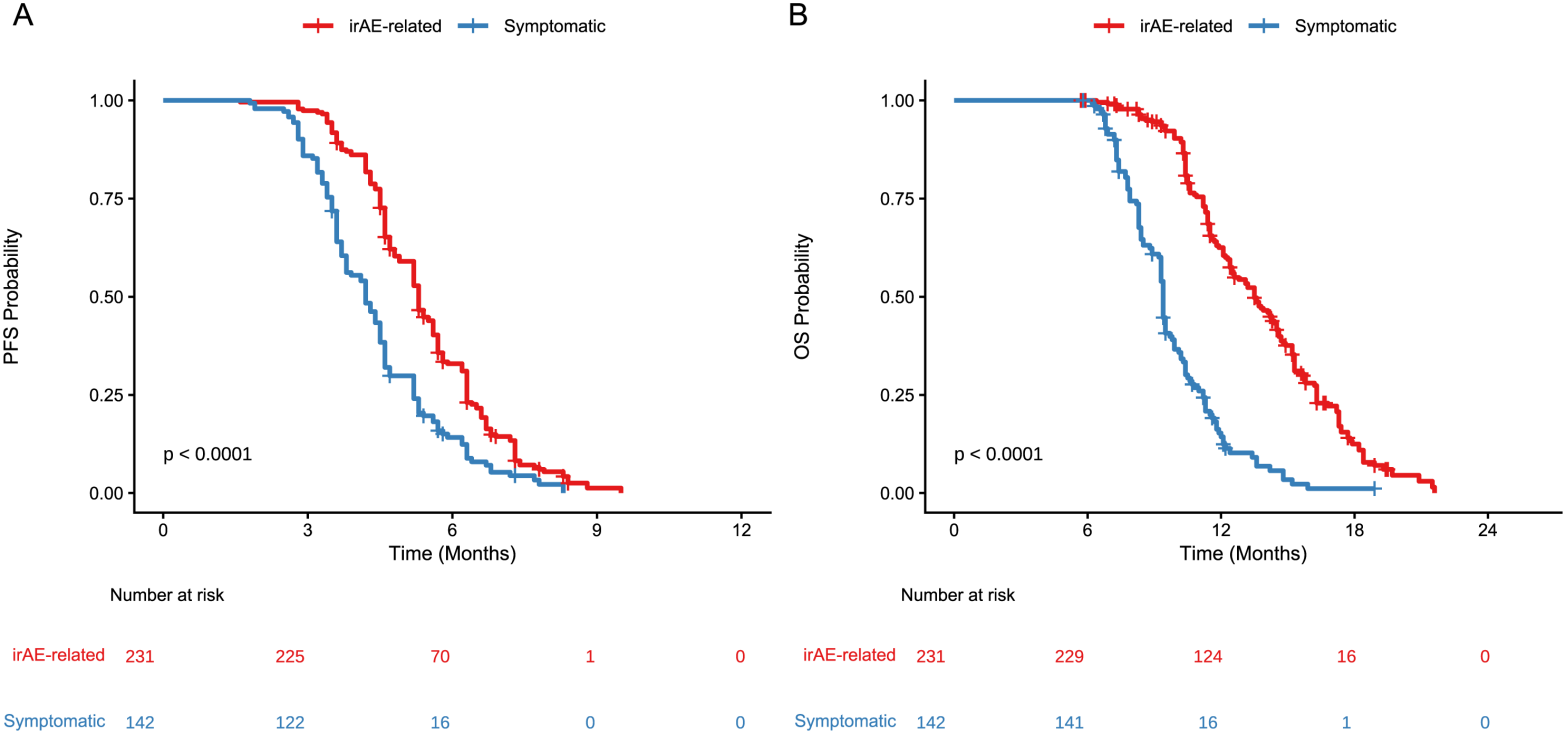
Survival outcomes according to the indication for corticosteroid use. **(A)** Progression-free survival (PFS) and **(B)** overall survival (OS) among patients receiving corticosteroids, stratified by indication (irAE-related vs symptomatic management). Survival curves were compared using the log-rank test; *p*-values are shown in the figure. irAE, immune-related adverse event; OS, overall survival; PFS, progression-free survival.

In indication-specific analyses among the 373 patients receiving corticosteroids, clinical outcomes diverged significantly according to steroid indication (**Figure 5**). Patients receiving CSs for irAE management had significantly longer PFS (median 5.3 months, 95% CI 5.2–5.6 vs. 4.2 months, 95% CI 3.8–4.5; p<0.0001; **Figure 5A**) and OS (median 13.5 months, 95% CI 12.5–14.5 vs. 9.4 months, 95% CI 9.3–9.7; p<0.0001; **Figure 5B**) than those receiving CSs for symptomatic management.

### Subgroup consistency and robustness analyses

**Figure 6:**
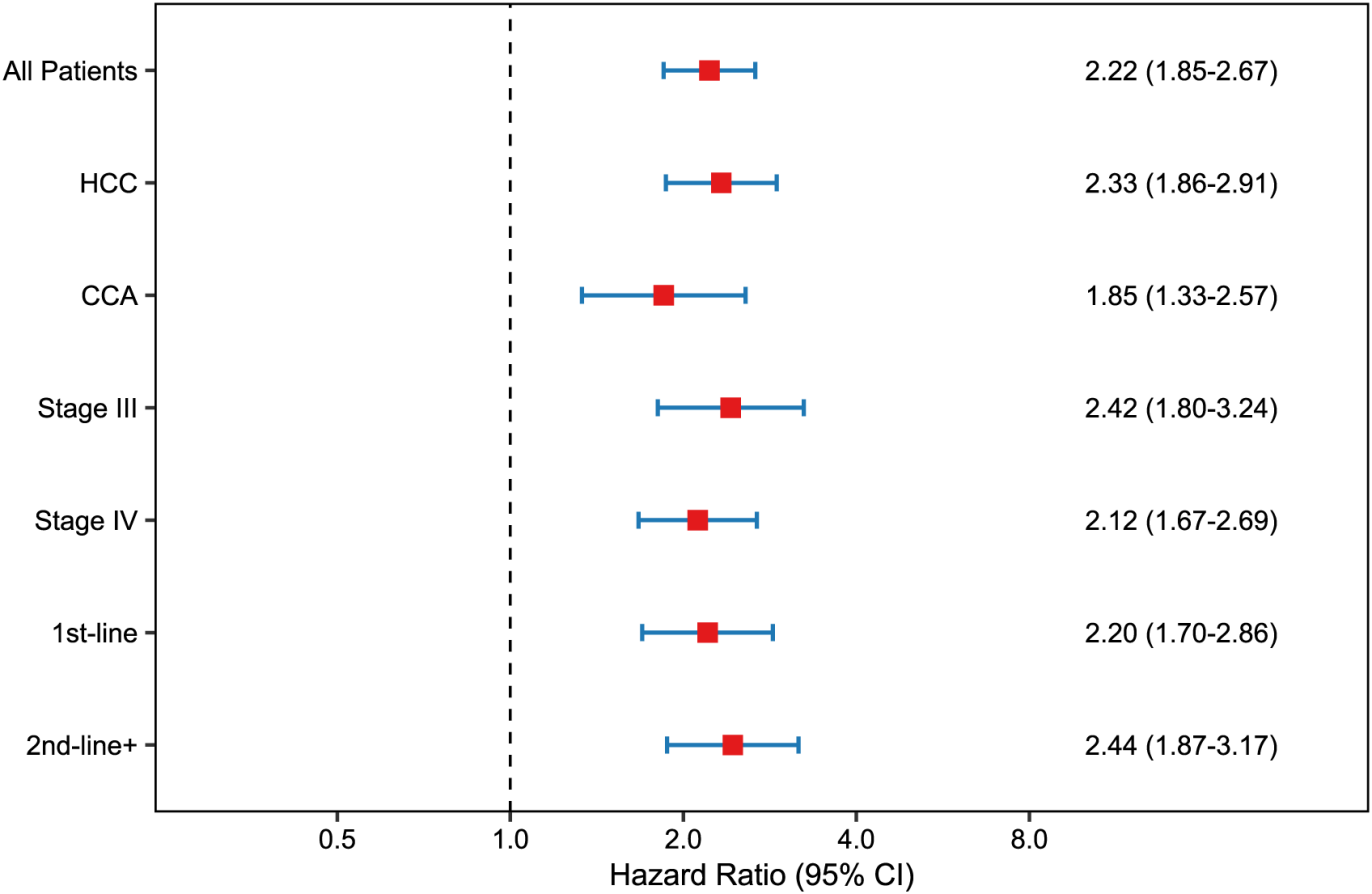
Subgroup analysis of the association between medication exposure and overall survival. Forest plot of hazard ratios (HRs) and 95% confidence intervals (CIs) for overall survival across pre-specified subgroups, derived from IPTW-adjusted Cox models. Interaction *p*-values are shown. CI, confidence interval; HR, hazard ratio.

Subgroup analyses confirmed that the association between combined ATB/CS exposure and poorer OS remained generally consistent across pre-specified strata (**Figure 6**). All hazard ratios were greater than 1.0 for subgroups defined by tumor type (HCC and CCA), TNM stage (Stage III and IV), and line of therapy (first line and second line or later), with no significant interactions observed between medication exposure and cancer subtype. Finally, in the 6-month landmark analysis, the survival disadvantage associated with symptomatic corticosteroid use and ATB exposure remained evident (p<0.0001; **Supplementary Figure 6**), supporting the robustness of the primary findings while partially mitigating concerns regarding immortal time bias.

## Discussion

In this retrospective cohort of 759 patients with advanced hepatobiliary malignancies, we demonstrate that concomitant exposure to antibiotics (ATBs) and corticosteroids (CSs) is independently and synergistically associated with inferior outcomes following immune checkpoint inhibitor (ICI) therapy. Although these medications did not significantly affect initial objective response rates, they were associated with impaired response durability and reduced overall survival (OS), suggesting a potential association with impaired durability rather than initial induction of antitumor immune responses. Importantly, indication-specific analyses revealed that CS use for immune-related adverse events (irAEs) was not associated with worse outcomes, whereas CS administration for symptomatic palliation identified a subgroup of patients with markedly poor prognosis. Collectively, these findings highlight the importance of context-dependent immune modulation and suggest that concomitant medication exposure may be associated with impaired maintenance of effective antitumor immunity during ICI therapy (24, 25).

The observed association between ATB exposure and poorer ICI outcomes is consistent with emerging evidence implicating the gut–liver axis as a key regulator of immunotherapy efficacy (27). In hepatobiliary malignancies, ATBs are frequently prescribed for biliary tract infections or spontaneous bacterial peritonitis; however, broad-spectrum antibiotics can disrupt gut microbial composition, leading to reduced diversity and depletion of taxa associated with effective antitumor immunity (28, 29). Such microbiome perturbations may impair antigen presentation, alter cytokine signaling, and disrupt metabolite-mediated immune regulation, potentially contributing to impaired systemic antitumor immune responses (30, 31). Prior translational and preclinical studies have suggested that antibiotic-associated dysbiosis may influence dendritic cell maturation and CD8^+^ T-cell priming, potentially affecting the persistence of antitumor immune responses. Notably, the stronger association with OS than with ORR in our study supports the hypothesis that microbiome disruption may preferentially influence the durability of immune responses rather than their initial induction (32).

The liver represents a uniquely tolerogenic immune organ enriched with Kupffer cells, regulatory T cells, myeloid-derived suppressor cells, and immunomodulatory cytokines that collectively shape antitumor immune responses (33, 34). In hepatobiliary malignancies, chronic inflammation and hepatic immune dysfunction may further contribute to an immunosuppressive tumor microenvironment. Emerging evidence suggests that disruption of the gut–liver axis by antibiotics may perturb microbial-derived immune signaling, alter hepatic immune homeostasis, and impair systemic antitumor immunity (35, 36). Nevertheless, these mechanisms remain biologically plausible hypotheses derived from prior translational and preclinical studies rather than direct mechanistic observations from the current retrospective clinical analysis (37, 38).

The role of corticosteroids in the context of immunotherapy remains complex and highly dependent on clinical indication. While early studies suggested a detrimental impact of baseline steroid use, our findings emphasize that the timing and purpose of CS administration are critical determinants of outcome. Patients receiving CSs for irAE management demonstrated significantly improved survival compared with those receiving CSs for symptomatic palliation. This observation supports the concept that irAEs may serve as a surrogate marker of robust immune activation, and that transient immunosuppression used to control toxicity does not necessarily abrogate antitumor efficacy. In contrast, CS use for symptomatic management often reflects advanced disease burden and a pro-inflammatory systemic state, as evidenced by higher baseline neutrophil-to-lymphocyte ratio (NLR) and adverse LIPI scores in our cohort (39, 40). Such an inflammatory milieu has been consistently associated with impaired immune responsiveness and poor clinical outcomes (41). Previous experimental studies have suggested that corticosteroids may suppress T-cell proliferation, reduce cytokine production, and alter the tumor immune microenvironment, thereby potentially limiting antitumor immune activity during ICI therapy.

A key strength of our study is the evaluation of the combined impact of ATB and CS exposure, which reflects a common yet understudied clinical scenario. Patients in the combined exposure group exhibited a markedly increased risk of mortality, suggesting a potential cumulative detrimental effect associated with concurrent exposure to multiple immune-modulating medications (27). Such combined exposure may reflect a clinically relevant state of compounded immune perturbation during immunotherapy, ultimately limiting the efficacy of ICIs (30, 31).

We acknowledge the inherent clinical heterogeneity between hepatocellular carcinoma (HCC) and cholangiocarcinoma (CCA), including differences in tumor biology, underlying liver disease, immune microenvironment, and therapeutic strategies. Nevertheless, both tumor types are increasingly managed with immune checkpoint inhibitors in real-world hepatobiliary oncology practice, supporting the clinical relevance of evaluating concomitant medication exposure across a broader ICI-treated hepatobiliary population. To address potential heterogeneity, cancer type and treatment-related variables were incorporated into the IPTW model, and subgroup analyses demonstrated generally consistent associations across both HCC and CCA cohorts. However, the present study was not designed to establish tumor-specific causal effects, and residual disease-specific confounding related to biliary complications, liver dysfunction, and treatment selection cannot be fully excluded. Future prospective disease-specific studies are warranted to validate these findings.

From a methodological perspective, retrospective analyses in this field are particularly vulnerable to selection bias and immortal time bias. We addressed these challenges through the application of IPTW to achieve robust covariate balance across key clinical parameters, including liver function (ALBI), tumor burden (LIPI/stage), and systemic inflammation (NLR) (42, 43). The persistence of the observed associations after weighting, together with confirmation in landmark and baseline-only sensitivity analyses, strengthens the robustness and internal validity of our findings.

Several limitations should be acknowledged. First, the retrospective design precludes direct characterization of microbiome alterations, which would provide deeper mechanistic insight into the observed associations between concomitant medications and immunotherapy outcomes. Second, although multiple clinicopathological variables were adjusted for using IPTW and multivariable models, residual confounding cannot be completely excluded. In particular, unmeasured factors such as infection severity, biliary complications, cachexia, dietary patterns, and specific antibiotic classes may have influenced both medication exposure and survival outcomes. Moreover, symptomatic corticosteroid use may partly represent a surrogate marker of advanced disease burden and systemic inflammatory status rather than a direct pharmacological effect alone. Third, treatment heterogeneity reflects real-world clinical practice and introduces additional complexity, particularly given the biological and therapeutic differences between hepatocellular carcinoma and cholangiocarcinoma. Fourth, because medication exposure was defined within a peri-ICI treatment window, time-related biases, including reverse causality and immortal time bias, cannot be entirely eliminated despite IPTW adjustment and landmark analyses. Retrospective treatment allocation and symptomatic medication use may also introduce treatment-selection bias and indication bias. To partially address these concerns, we performed additional baseline-only sensitivity analyses restricted to pre-ICI exposure, which yielded generally consistent findings and supported the robustness of the primary results. In addition, tumor response was evaluated using RECIST v1.1 rather than immune-specific criteria such as iRECIST or mRECIST, which may have limited the ability to fully capture atypical immunotherapy response patterns, including pseudoprogression. Finally, the retrospective design limited detailed time-varying characterization of medication exposure. We were unable to comprehensively model the sequence, cumulative dose intensity, duration, administration route, and dynamic timing of antibiotic and corticosteroid exposure, particularly within the combined exposure group. Consequently, the present findings should be interpreted as reflecting the overall clinical impact of concomitant medication exposure rather than the effects of specific pharmacological subtypes or dosing patterns. Future prospective studies incorporating longitudinal microbiome profiling, immune phenotyping, and time-dependent exposure modeling are warranted to validate these findings and further clarify the underlying biological mechanisms.

In conclusion, concomitant antibiotic exposure and corticosteroid use for non-irAE indications were associated with inferior survival outcomes in patients with hepatobiliary malignancies receiving immune checkpoint inhibitors. These findings suggest that concomitant medication exposure may influence the durability of immunotherapy responses in real-world clinical settings. Clinically, this highlights the importance of judicious antibiotic use and careful consideration of corticosteroid indications during immunotherapy. Importantly, corticosteroids administered for irAE management were not associated with diminished therapeutic efficacy in the present cohort. Future prospective studies incorporating longitudinal microbiome profiling and immune characterization are warranted to validate these observations and further clarify the underlying biological mechanisms.

## Data Availability

The data analyzed in this study is subject to the following licenses/restrictions: The datasets are not publicly available due to institutional privacy regulations and ethical restrictions involving patient confidentiality. Requests to access these datasets should be directed to JR, 869491533@qq.com.
