# Supplementary material for: Antibiotic exposure and indication-specific corticosteroid use differentially modulate outcomes of immune checkpoint inhibitor therapy in hepatobiliary malignancies
Source: Front Immunol. 2026 Jun 29;17:1873839. doi: 10.3389/fimmu.2026.1873839 (PMC13357655; doi:10.3389/fimmu.2026.1873839)
Supplement: Supplementary file 1 [file Image1.pdf]

Supplementary Material

Supplementary Figures

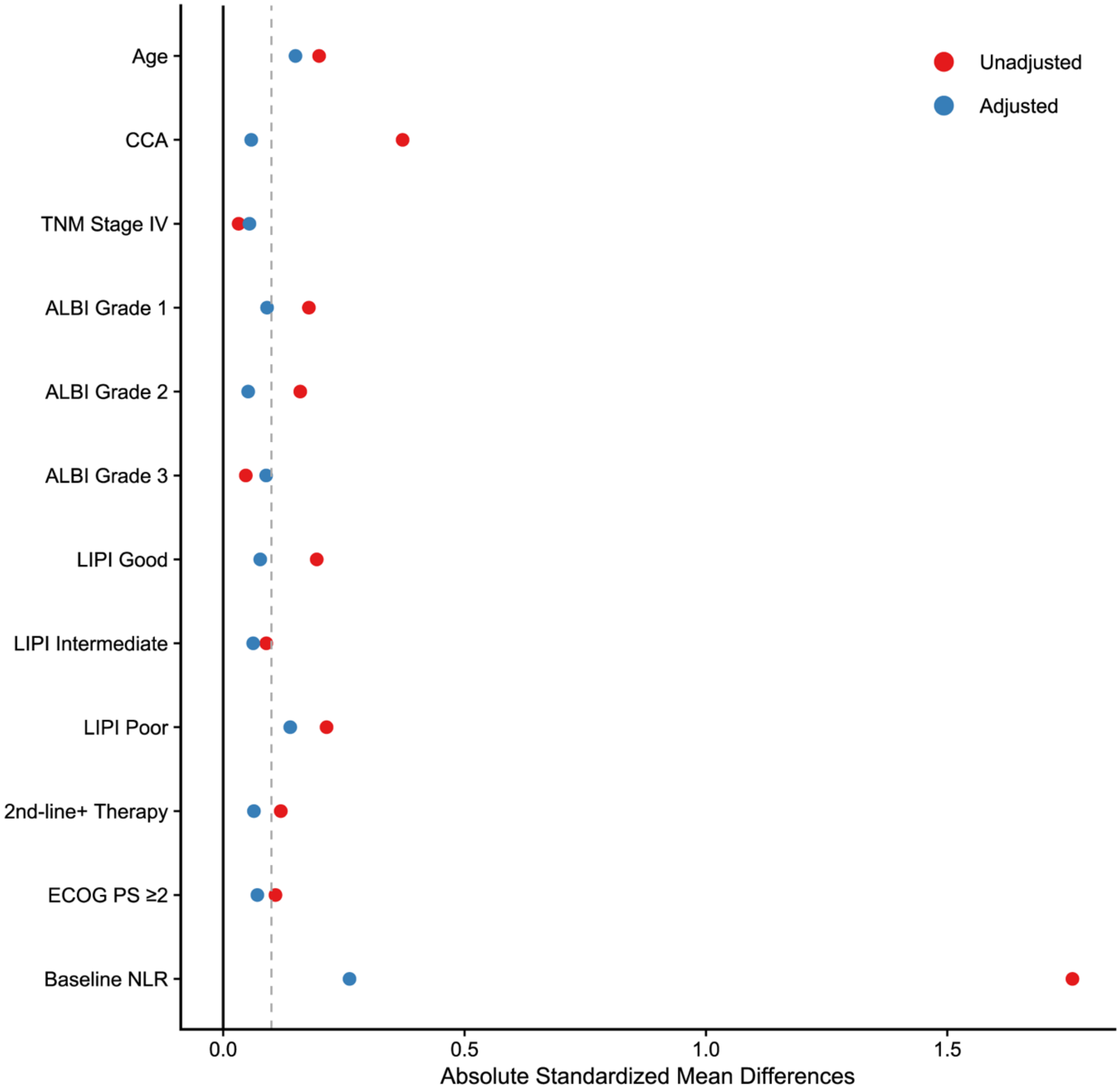

Supplementary Figure S1. Covariate balance before and after IPTW.

Love plot showing absolute standardized mean differences (SMDs) for baseline covariates. The maximum SMD across treatment groups is presented. Blue dots represent the IPTW-adjusted cohort, demonstrating improved balance (SMD <0.1).

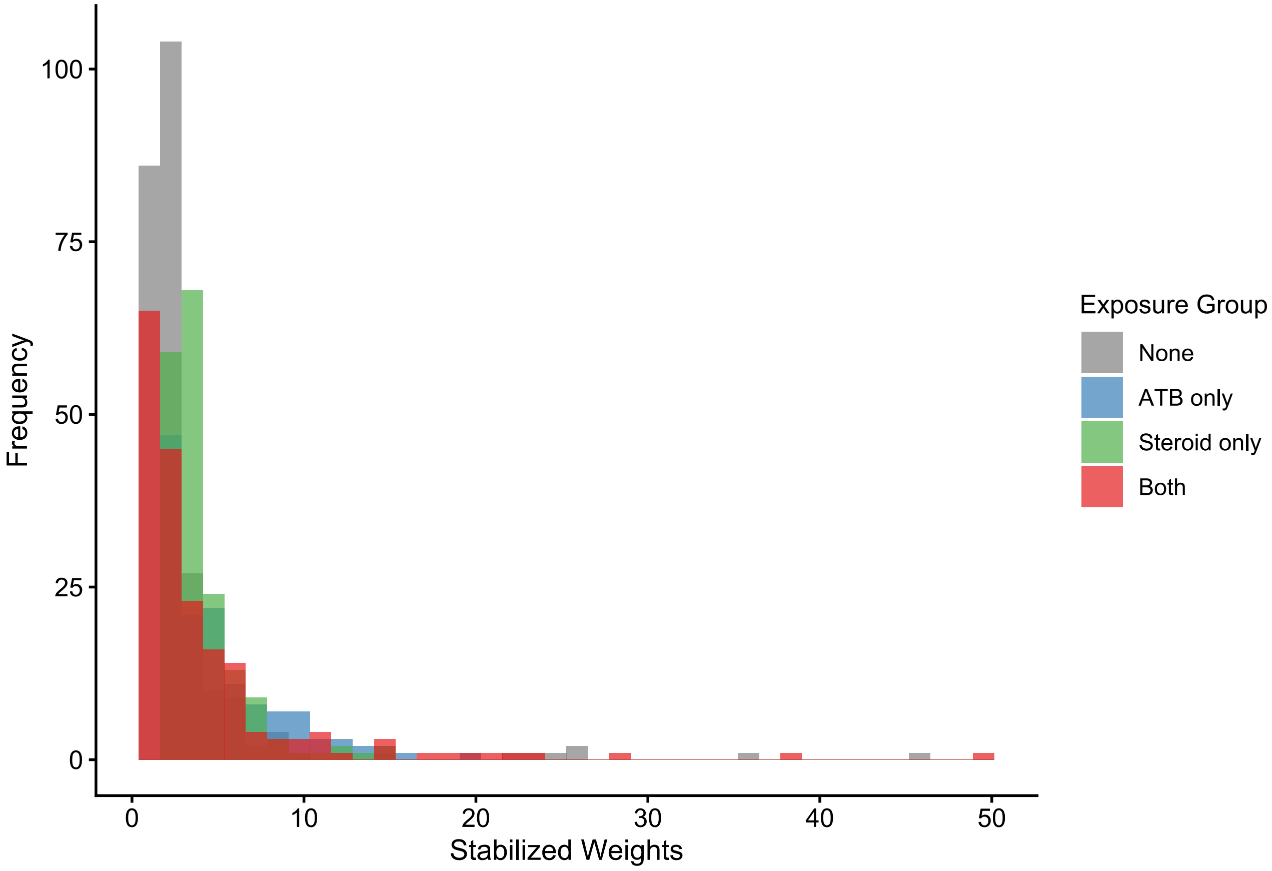

Supplementary Figure S2. Distribution of stabilized inverse probability of treatment weights (IPTW).

Histogram showing the distribution of stabilized IPTW weights across the study population after propensity score weighting. Most weights were concentrated within a low-to-moderate range, with no evidence of excessive extreme weights, supporting acceptable model stability and positivity assumptions for the weighted analyses. IPTW, inverse probability of treatment weighting.

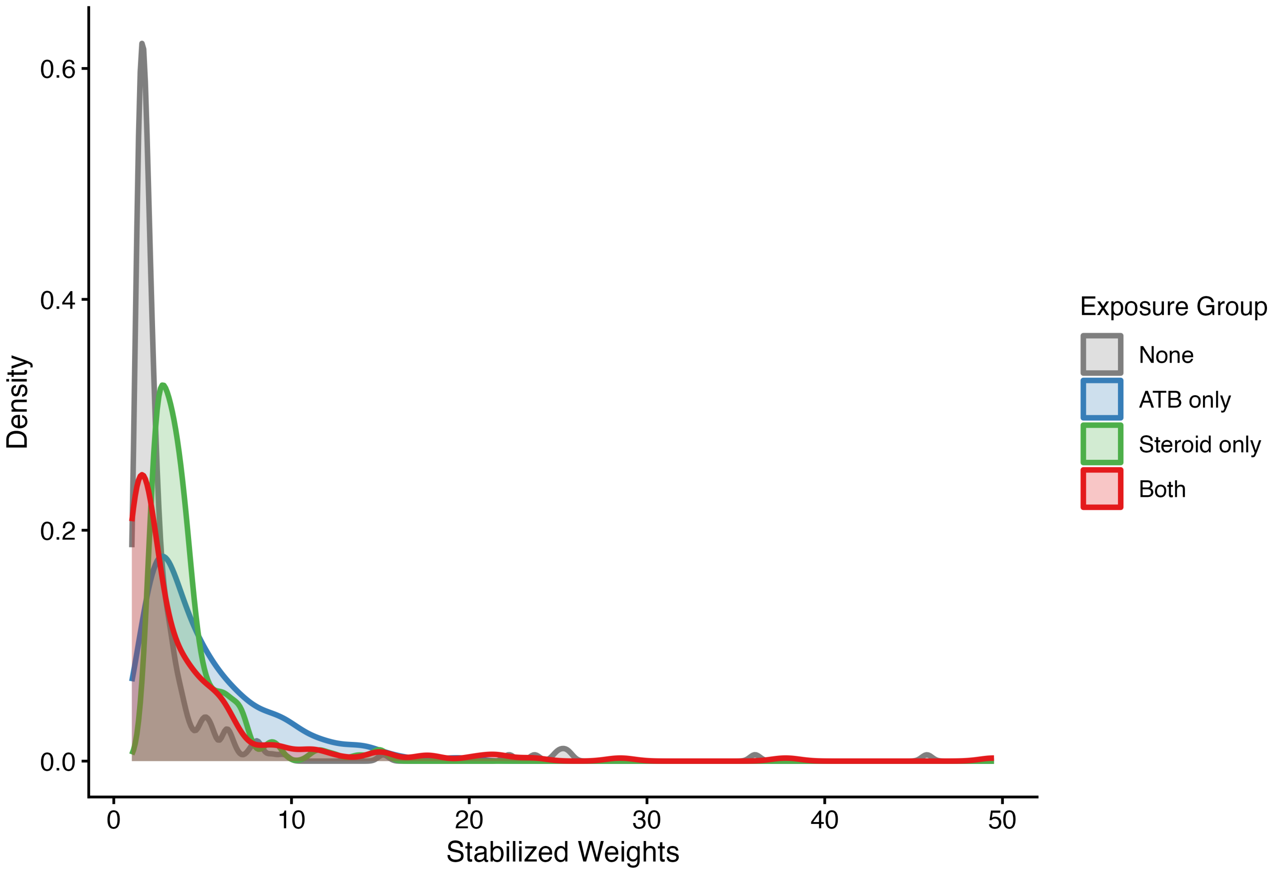

Supplementary Figure S3. Density distribution of stabilized IPTW weights across exposure groups.

The distribution of stabilized inverse probability treatment weights demonstrated acceptable overlap across exposure groups without evidence of severe positivity violations or extreme instability.

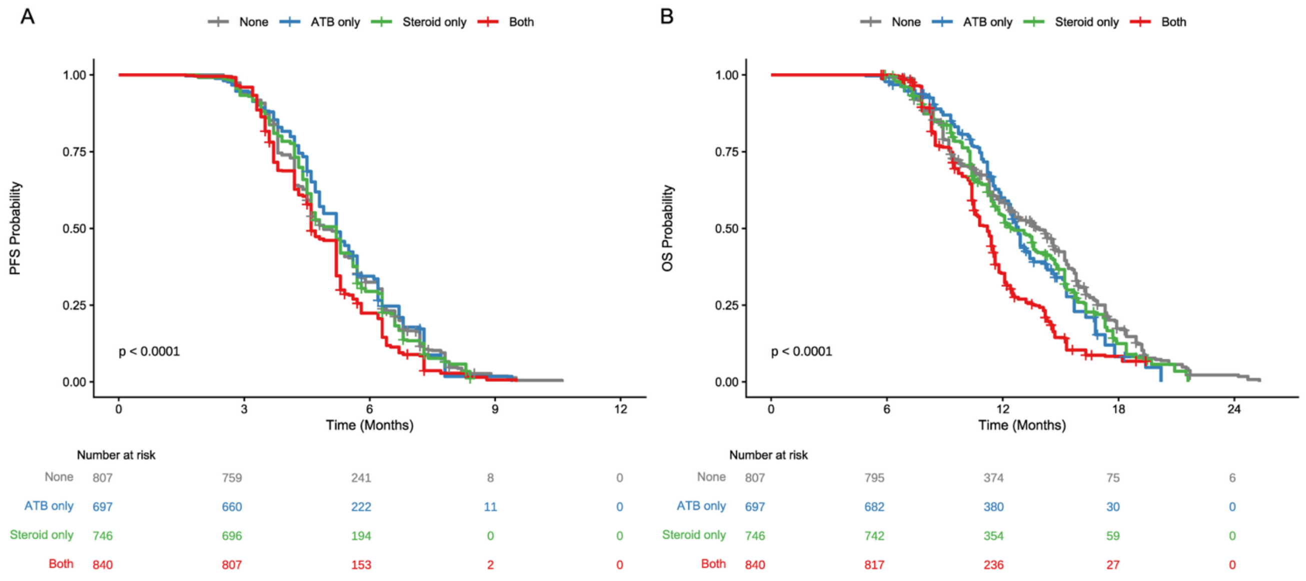

Supplementary Figure S4. IPTW-adjusted survival analysis.

(A) Kaplan–Meier curves for progression-free survival (PFS) after inverse probability of treatment weighting (IPTW) to balance baseline covariates.

(B) Kaplan–Meier curves for overall survival (OS) after IPTW adjustment to balance baseline characteristics.

The survival disadvantage associated with antibiotic (ATB) and corticosteroid exposure remained robust in the weighted pseudo-population ($p < 0.0001$).

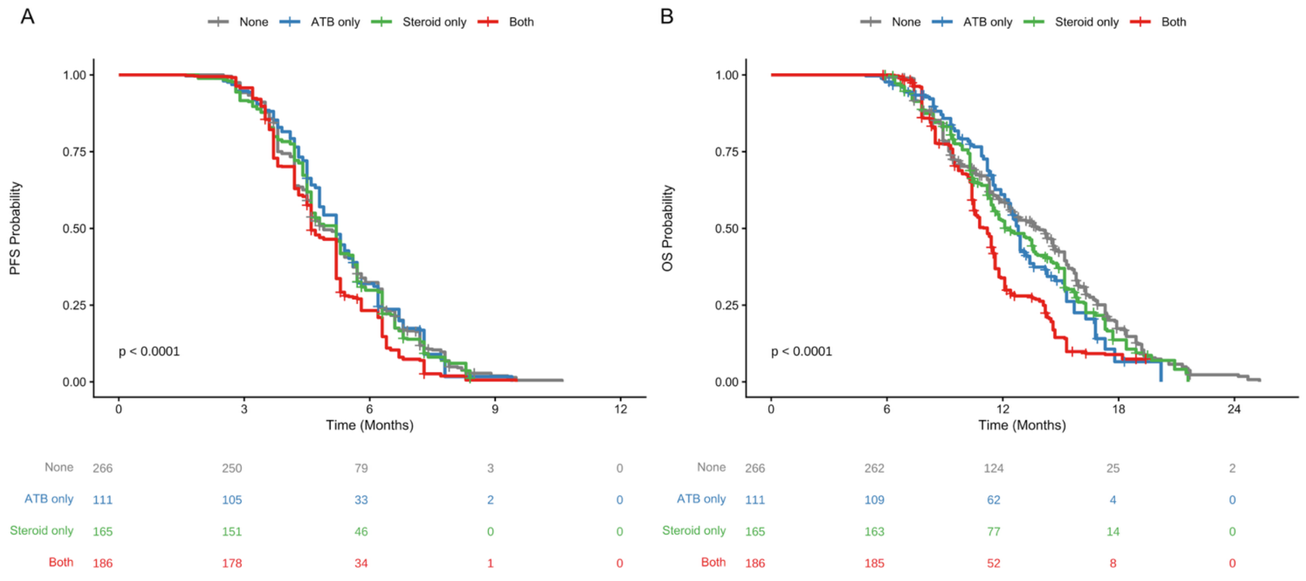

Supplementary Figure S5. Baseline-only IPTW-adjusted survival analysis according to medication exposure.

Kaplan–Meier curves for progression-free survival (PFS) (A) and overall survival (OS) (B) after restricting exposure definitions to baseline medication use before immune checkpoint inhibitor (ICI) initiation. Patients were categorized into four groups according to antibiotic (ATB) and corticosteroid (CS) exposure status: None, ATB only, Steroid only, and Both. Inverse probability of treatment weighting (IPTW) based on multinomial propensity scores was applied to balance baseline covariates across groups. Risk tables are shown below each panel.

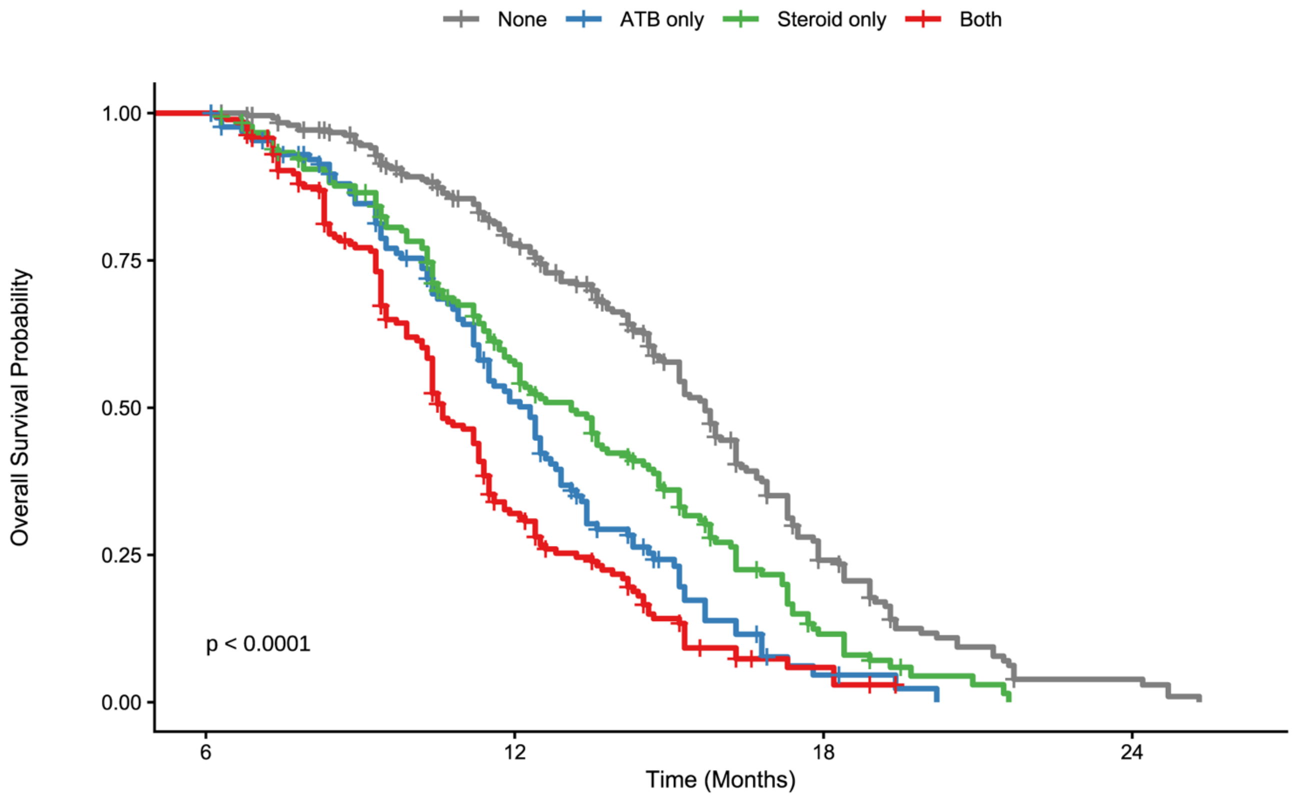

Number at risk

None	247	160	36	4
ATB only	130	58	3	0
Steroid only	182	91	13	0
Both	188	49	4	0

Supplementary Figure S6. Landmark analysis of overall survival at 6 months.

Landmark analysis of overall survival excluding patients who died or were censored within 6 months after initiation of immune checkpoint inhibitor (ICI) therapy to reduce potential immortal time bias. The survival differences among the four exposure groups remained statistically significant ($p < 0.0001$), confirming the long-term association between medication exposure and survival outcomes.
