# Supplementary material for: Antibiotic exposure and indication-specific corticosteroid use differentially modulate outcomes of immune checkpoint inhibitor therapy in hepatobiliary malignancies
Source: Front Immunol. 2026 Jun 29;17:1873839. doi: 10.3389/fimmu.2026.1873839 (PMC13357655; doi:10.3389/fimmu.2026.1873839)
Supplement: Supplementary file 2 [file DataSheet1.docx]

Supplementary Material

**Supplementary Methods**

**1. Propensity Score Weighting and Balance Diagnostics**

To control for confounding and baseline imbalances among the four exposure groups, inverse probability of treatment weighting (IPTW) was performed using the WeightIt package in R. Propensity scores were estimated using a multinomial logistic regression model incorporating predefined baseline covariates, including age, cancer type, TNM stage, ECOG performance status, ALBI grade, LIPI score, line of therapy, treatment regimen, and baseline neutrophil-to-lymphocyte ratio.

The average treatment effect (ATE) estimand was applied. Stabilized weights were generated to improve precision and reduce the impact of extreme weights. Weight distributions were visually inspected using density and histogram plots to assess model stability, positivity assumptions, and common support across treatment groups (Supplementary Figure S2). Propensity score overlap across exposure groups was evaluated graphically to further assess common support (Supplementary Figure S3).

Covariate balance before and after weighting was assessed using absolute standardized mean differences (SMDs), with an SMD <0.1 considered indicative of adequate balance. Balance diagnostics were visualized using Love plots generated with the cobalt package (Supplementary Figure S1).

**2. Landmark Analysis**

To reduce potential immortal time bias, a landmark analysis was performed at 6 months after initiation of immune checkpoint inhibitors. Only patients who were alive and not censored before the landmark time point were included.

This analysis was used to validate the robustness of the association between medication exposure and overall survival.

**3. Software and Statistical Packages**

All statistical analyses were conducted using R software (version 4.3.2; R Foundation for Statistical Computing, Vienna, Austria).

Key packages included:

- WeightIt for IPTW estimation
- cobalt for covariate balance assessment
- survival and survminer for time-to-event analyses
- ggplot2 for data visualization
