# Supplementary material for: Antibiotic exposure and indication-specific corticosteroid use differentially modulate outcomes of immune checkpoint inhibitor therapy in hepatobiliary malignancies
Source: Front Immunol. 2026 Jun 29;17:1873839. doi: 10.3389/fimmu.2026.1873839 (PMC13357655; doi:10.3389/fimmu.2026.1873839)
Supplement: Supplementary file 3 [file Table1.docx]

Supplementary Material

Supplementary Tables

**Supplementary Table S1. Baseline-only multivariable Cox proportional hazards analysis for overall survival.**

| **Variable** | **Level** |  | **HR(95%CI)** | ***P* value** |
| --- | --- | --- | --- | --- |
| **Medication** | None | Ref | 2.02 (1.52–2.68) |  |
|  | ATB only |  | 1.95 (1.53–2.50) | **<0.001** |
|  | Steroid only |  | 3.24 (2.39–4.40) | **<0.001** |
|  | Both |  |  | **<0.001** |
| **Tumor Type** | HCC | Ref | 3.77 (2.60–5.47) |  |
|  | CCA |  |  | **<0.001** |
| **TNM Stage** | Stage III | Ref | 1.30 (1.09–1.56) |  |
|  | Stage IV |  |  | **0.0043** |
| **ALBI Grade** | G1 | Ref | 2.01 (1.62–2.49) |  |
|  | G2 |  | 8.53 (6.11–11.91) | **<0.001** |
|  | G3 |  |  | **<0.001** |
| **LIPI Score** | Good | Ref | 2.38 (1.91–2.97) |  |
|  | Intermediate |  | 6.76 (5.10–8.97) | **<0.001** |
|  | Poor |  |  | **<0.001** |
| **Therapy Line** | 1st line | Ref | 2.03 (1.64–2.50) |  |
|  | 2nd+ line |  |  | **<0.001** |
| **ECOG PS** | 0-1 | Ref | 1.09 (0.88–1.36) |  |
|  | ≥2 |  | 1.07 (1.03–1.11) | 0.4312 |
| **NLR** |  |  |  | **0.0013** |
| **Regimen** | PD-1 mono | Ref | 0.44 (0.33–0.58) |  |
|  | PD-1 + TKI |  | 0.46 (0.32–0.68) | **<0.001** |
|  | PD-1 + Chemo |  | 0.83 (0.60–1.14) | **<0.001** |
|  | PD-1 + Radio |  | 1.135665 | 0.2383 |

Multivariable Cox regression analysis evaluating the association between baseline-only antibiotic and corticosteroid exposure and overall survival after restricting medication exposure to the pre-ICI period. Covariates included tumor type, TNM stage, ALBI grade, LIPI score, treatment line, ECOG performance status, treatment regimen, and baseline neutrophil-to-lymphocyte ratio (NLR). Hazard ratios (HRs) with 95% confidence intervals (CIs) are reported.

**Supplementary Table S2. Best overall response and clinical efficacy across medication exposure groups.**

| **Characteristic** | **None** N = 251 | **ATB only** N = 135 | **Steroid only** N = 183 | **Both** N = 190 | ***p*-value** |
| --- | --- | --- | --- | --- | --- |
| **Best Overall Response** |  |  |  |  |  |
| CR | 0 (0%) | 0 (0%) | 0 (0%) | 0 (0%) |  |
| PR | 36 (14%) | 14 (10%) | 15 (8.2%) | 22 (12%) |  |
| SD | 152 (61%) | 84 (62%) | 110 (60%) | 110 (58%) |  |
| PD | 63 (25%) | 37 (27%) | 58 (32%) | 58 (31%) |  |
| **ORR** |  |  |  |  | 0.3 |
| Non-responded | 215 (86%) | 121 (90%) | 168 (92%) | 168 (88%) |  |
| Responded | 36 (14%) | 14 (10%) | 15 (8.2%) | 22 (12%) |  |
| **DCR** |  |  |  |  | 0.4 |
| Controlled | 188 (75%) | 98 (73%) | 125 (68%) | 132 (69%) |  |
| Not-controlled | 63 (25%) | 37 (27%) | 58 (32%) | 58 (31%) |  |

Comparison of objective response rate (ORR) and disease control rate (DCR) across groups. ORR is defined as the sum of complete and partial responses, and DCR as the sum of complete response, partial response, and stable disease. *p*-values were calculated using Fisher’s exact test.

**Supplementary Table S3. Multivariable Cox proportional hazards analysis for OS and PFS.**

| **Variable** | **Level** | **OS: HR (95% CI)** | **OS: *p*-value** | **PFS: HR (95% CI)** | **PFS: *p*-value** |
| --- | --- | --- | --- | --- | --- |
| **Medication Exposure** | None | 1.00 (Ref.) | - | 1.00 (Ref.) | - |
|  | ATB only | 1.97 (1.49–2.59) | **< 0.001** | 1.18 (0.93–1.49) | 0.175 |
|  | Steroid only | 1.94 (1.53–2.45) | **< 0.001** | 1.08 (0.88–1.34) | 0.459 |
|  | Both | **3.09 (2.31–4.13)** | **< 0.001** | **1.30 (1.02–1.65)** | **0.037** |
| **Tumor Type** | HCC | 1.00 (Ref.) | - | 1.00 (Ref.) | - |
|  | CCA | 3.38 (2.36–4.85) | **< 0.001** | 1.78 (1.30–2.45) | **< 0.001** |
| **TNM Stage** | Stage III | 1.00 (Ref.) | - | 1.00 (Ref.) | - |
|  | Stage IV | 1.25 (1.05–1.49) | **0.014** | 1.22 (1.04–1.44) | **0.015** |
| **ALBI Score** | Grade 1 | 1.00 (Ref.) | - | 1.00 (Ref.) | - |
|  | Grade 2 | 2.10 (1.70–2.58) | **< 0.001** | 1.01 (0.84–1.22) | 0.876 |
|  | Grade 3 | 8.97 (6.49–12.40) | **< 0.001** | 0.88 (0.68–1.13) | 0.313 |
| **LIPI Score** | Good | 1.00 (Ref.) | - | 1.00 (Ref.) | - |
|  | Intermediate | 2.51 (2.02–3.11) | **< 0.001** | 1.34 (1.11–1.61) | **0.002** |
|  | Poor | 7.14 (5.44–9.38) | **< 0.001** | 2.45 (1.93–3.10) | **< 0.001** |
| **Therapy Line** | 1st line | 1.00 (Ref.) | - | 1.00 (Ref.) | - |
|  | 2nd+ line | 1.99 (1.63–2.43) | **< 0.001** | 1.01 (0.85–1.21) | 0.889 |
| **ECOG PS** | 0–1 | 1.00 (Ref.) | - | 1.00 (Ref.) | - |
|  | ≥ 2 | 1.13 (0.91–1.40) | 0.272 | 1.44 (1.19–1.75) | **< 0.001** |
| **Baseline NLR** | (Continuous) | 1.08 (1.04–1.12) | **< 0.001** | 1.04 (1.00–1.08) | **0.030** |
| **Treatment Regimen** | PD-1 mono | 1.00 (Ref.) | - | 1.00 (Ref.) | - |
|  | PD-1+TKI | 0.46 (0.35–0.60) | **< 0.001** | 0.41 (0.32–0.53) | **< 0.001** |
|  | PD-1+Chemo | 0.56 (0.39–0.81) | **0.002** | 0.32 (0.23–0.45) | **< 0.001** |
|  | PD-1+Radio | 0.91 (0.67–1.25) | 0.568 | 0.79 (0.58–1.06) | 0.114 |

Multivariable Cox regression models reporting hazard ratios (HRs), 95% confidence intervals (CIs), and p-values for overall survival (OS) and progression-free survival (PFS). All models were adjusted for baseline covariates listed in Table 1. Figure 4 in the main text presents the OS model graphically, whereas this table includes results for both OS and PFS endpoints.

Abbreviations: ALBI, Albumin-Bilirubin; CI, confidence interval; HR, hazard ratio; LIPI, Lung Immune Prognostic Index; NLR, neutrophil-to-lymphocyte ratio; OS, overall survival; PFS, progression-free survival; Ref., reference.
